# SSGJ-608 in moderate-to-severe plaque psoriasis: a multicenter, randomized, open-label, phase 3 study

**DOI:** 10.3389/fimmu.2026.1810418

**Published:** 2026-06-09

**Authors:** Lin Cai, Jing Chen, Liming Wu, Xinsuo Duan, Guoqiang Zhang, Yumei Li, Litao Zhang, Lanying Qin, Tongxiang Zeng, Xiaohua Wang, Jinyan Wang, Kun Huang, Hong Ren, Lunfei Liu, Yangfeng Ding, Yong Cui, Yunyun Shan, Jianyun Lu, Xiaohua Tao, Rixin Chen, Yin Tu, Min Yan, Xiaohong Zhu, Na Qiao, Zudong Meng, Yu Wang, Fang Cheng, Yanxia Yuan, Jianjian Zhu, Xiaoping Hu, Shuping Guo, Xiujuan Xia, Xiaoyong Man, Zhouwei Wu, Xuejun Chen, Guanzhi Chen, Yingxia He, Dong Lv, Yanyan Feng, Danqi Deng, Songmei Geng, Qing Guo, Wenli Feng, Xiulan Zhu, Yongjun Liu, Bingjiang Lin, Rushan Xia, Chunshui Yu, Juanli Fan, Mingkai Ji, Tiechi Lei, Wenlin Yang, Meiping Yang, Ying Gao, Weiquan Li, Meiying Jiang, Jing Lou, Yanli Liu, Cheng Zhou, Jianzhong Zhang

**Affiliations:** 1 Peking University People’s Hospital, Beijing, China; 2 The Third Xiangya Hospital of Central South University, Changsha, Hunan, China; 3 Hangzhou First People’s Hospital, Hangzhou, Zhejiang, China; 4 Affiliated Hospital of Chengde Medical University, Chengde, Hebei, China; 5 The First Hospital of Hebei Medical University, Shijiazhuang, Hebei, China; 6 Affiliated Hospital of Jiangsu University, Zhenjiang, Jiangsu, China; 7 Affiliated Hospital of Tianjin Academy of Traditional Chinese Medicine, Tianjin, China; 8 Cangzhou People’s Hospital, Cangzhou, Hebei, China; 9 Jingzhou Central Hospital, Jingzhou, Hubei, China; 10 Dermatology Hospital of Southern Medical University, Guangzhou, China; 11 Ningbo Second Hospital (Huamei Hospital), University of Chinese Academy of Sciences, Ningbo, Zhejiang, China; 12 The First Affiliated Hospital of Chongqing Medical University, Chongqing, China; 13 The First People’s Hospital of Lianyungang, Lianyungang, Jiangsu, China; 14 The Fourth Affiliated Hospital, Zhejiang University School of Medicine, Yiwu, Zhejiang, China; 15 Shanghai Dermatology Hospital, Shanghai, China; 16 China-Japan Friendship Hospital, Beijing, China; 17 Hangzhou Third People’s Hospital, Hangzhou, Zhejiang, China; 18 Zhejiang Provincial People’s Hospital, Hangzhou, Zhejiang, China; 19 The First People’s Hospital of Nanyang, Nanyang, Henan, China; 20 The First Affiliated Hospital of Kunming Medical University, Yunnan, Kunming, China; 21 Shengli Oilfield Central Hospital, Dongying, Shandong, China; 22 Wuxi Second People’s Hospital, Wuxi, Jiangsu, China; 23 The First People’s Hospital of Qujing, Qujing, Yunnan, China; 24 Shiyan People’s Hospital, Shiyan, Hubei, China; 25 The Affiliated Hospital of Guizhou Medical University, Guiyang, Guizhou, China; 26 Xingtai People’s Hospital, Xingtai, Hebei, China; 27 The First People’s Hospital of Changzhou, Changzhou, Jiangsu, China; 28 The First People’s Hospital of Changde, Changde, Hunan, China; 29 Peking University Shenzhen Hospital, Shenzhen, China; 30 The First Hospital of Shanxi Medical University, Taiyuan, Shaanxi, China; 31 Yantai Yuhuangding Hospital, Yantai, Shandong, China; 32 The Second Affiliated Hospital, Zhejiang University School of Medicine, Hangzhou, Zhejiang, China; 33 Shanghai General Hospital, Shanghai, China; 34 Sichuan Provincial People’s Hospital, Chengdu, Sichuan, China; 35 The Affiliated Hospital of Qingdao University, Qingdao, Shandong, China; 36 Panjin Liaoyou Gem Flower Hospital, Panjin, Liaoning, China; 37 Yancheng First People’s Hospital, Yancheng, Jiangsu, China; 38 Chengdu Second People’s Hospital, Chengdu, Sichuan, China; 39 The Second Affiliated Hospital of Kunming Medical University, Kunming, Yunnan, China; 40 The Second Affiliated Hospital of Xi’an Jiaotong University, Xi’an, Shaanxi, China; 41 Sun Yat-sen Memorial Hospital of Sun Yat-sen University, Guangzhou, Guangdong, China; 42 The Second Hospital of Shanxi Medical University, Taiyuan, Shanxi, China; 43 The Second People’s Hospital of Changzhi, Changzhi, Shanxi, China; 44 The Second Affiliated Hospital of Heilongjiang University of Chinese Medicine, Harbin, Heilongjiang, China; 45 The First Affiliated Hospital of Ningbo University, Ningbo, Zhejiang, China; 46 Jiangyin Hospital of Traditional Chinese Medicine, Jiangyin, Jiangsu, China; 47 Suining Central Hospital, Suining, Sichuan, China; 48 Yuncheng Central Hospital of Shanxi Province, Yuncheng, Shanxi, China; 49 The Second Affiliated Hospital of Xiamen Medical College, Xiamen, Fujian, China; 50 Renmin Hospital of Wuhan University, Wuhan, Hubei, China; 51 The Second Affiliated Hospital of Guangzhou Medical University, Guangzhou, Guangdong, China; 52 Jiangxi Provincial People’s Hospital, Nanchang, Jiangxi, China; 53 Wuhan Central Hospital, Wuhan, Hubei, China; 54 Yuebei People’s Hospital, Shaoguan, Guangdong, China; 55 The Second Affiliated Hospital of Nanchang University, Nanchang, Jiangxi, China; 56 Sunshine Guojian Pharmaceutical (Shanghai) Co., Ltd., Shanghai, China

**Keywords:** clinical trial, efficacy and safety, IL-17, plaque psoriasis, SSGJ-608

## Abstract

**Background:**

SSGJ-608 is an anti-interleukin-17A monoclonal antibody with high specificity and high affinity and has shown promising efficacy in treatment of moderate-to-severe psoriasis in preliminary trials.

**Objective:**

This multicenter, randomized, open-label, phase 3 trial aimed to further evaluate SSGJ-608 at different dosing intervals (80mg every two weeks and 160mg every four weeks) in patients with moderate-to-severe plaque psoriasis.

**Methods:**

A total of 770 patients with moderate to severe plaque psoriasis were randomly assigned (1:1) to receive subcutaneous injections of 80mg of SSGJ-608 every two weeks (Q2W) after a starting dose of 160mg at week 0(608A group), or 160mg of SSGJ-608 every four weeks (Q4W) (608 B group) for 12 weeks. Efficacy was assessed by PASI75 and sPGA 0 or 1 response rates at week 12 as co-primary endpoints, and proportion of patients who achieved PASI90, PASI100 or sPGA score of 0 at week 12 as secondary endpoints. The safety profile was also evaluated.

**Results:**

At week12, the proportions of patients achieving PASI75 (92.7% vs. 95.1%) and sPGA 0/1 (80.3% vs. 79.0%) were comparable between the two SSGJ-608 dose regimens. The PASI90, PASI100 and sPGA 0 response rates were 81.0% vs.82.3%, 49.4% vs. 47.5%, and 49.4% vs.47.3% in the 608A group and the 608B group, respectively. In the subgroup of patients previously treated with anti-IL-17 therapy, SSGJ-608 also achieved high clinical response rates at week12. The most common TEAEs were hypertriglyceridemia, upper respiratory tract infection, hyperuricemia, increased alanine aminotransferase and hypercholesterolemia. Both treatment groups demonstrated a favorable safety profile and no new safety signals were identified.

**Conclusions:**

SSGJ-608 was highly effective for treating patients with moderate-to-severe plaque psoriasis at 80mg Q2W and 160mg Q4W in a larger population, especially in patients previously treated with anti-IL-17 therapy, and exhibited a favorable tolerability profile in Chinese patients with moderate-to-severe plaque psoriasis.

**Clinical trial registration:**

https://clinicaltrials.gov/, identifier NCT06299982.

## Introduction

Psoriasis is a chronic inflammatory disease characterized by sharply demarcated erythematous and scaly skin lesions, and accompanied by systemic manifestations, including psoriatic arthritis, metabolic disorders, cardiovascular disease, malignancies, chronic kidney disease (CKD), psychiatric illness, and inflammatory bowel disease (IBD) (1, 2). Psoriasis can result in profound functional, psychological and social morbidity, and has been linked to reduced levels of employment and income (3–5). Patients with psoriasis face significant health and quality of life challenges, and may require lifelong affordable treatment due to the chronic-relapsing nature of the disease (6, 7).

Multiple approved biologic therapies, including those inhibiting tumor necrosis factor (TNF), interleukin (IL)-12/23, IL-17, and IL-23, are effective treatments for moderate to severe psoriasis (8, 9). Biologics targeting IL-17, such as secukinumab and ixekizumab, neutralize IL-17A directly, blocking its binding to receptors, and have a rapid onset, typically showing significant improvement in skin lesions within 2 to 4 weeks (10). However, some biologics showed a decreased efficacy in a patient who initially responded well to a treatment and lose effectiveness over time, and the maintenance of response of IL-17A inhibitors might not be as robust as that of IL-23 inhibitors (10–17). Besides, depression and suicidal behavior have been reported with brodalumab use, of which not only impaired patients’ quality of life but may also be life-threatening. Consequently, achieving and sustaining complete skin clearance to ensure long-term efficacy, while simultaneously improving quality of life and preventing complications, represents the fundamental objective of contemporary psoriasis management.

Although there is substantial clinical experience with IL-17-targeted therapies for psoriasis, the initial 12-week induction treatment period plays a particularly crucial role in achieving significant clinical improvement. SSGJ-608 (Sunshine Guojian Pharmaceutical (Shanghai) Co., Ltd.) is a recombinant humanized IgG1 monoclonal antibody that targets human IL-17A with high specificity and high affinity. Previous studies have revealed the potential efficacy and tolerability of subcutaneous doses of 80–160 mg SSGJ-608 in psoriasis, and SSGJ-608 is the first IL-17A agent which has the potential to be administrated at a dosing interval of 8 weeks during the maintenance period. Here, we conducted a multicenter, randomized, open-label, phase 3 clinical trial to evaluate the efficacy and safety of SSGJ-608 at 80mg Q2W (with a 160mg starting dose at Week 0) and 160mg Q4W in a larger population in Chinese patients with moderate-to-severe plaque psoriasis.

## Methods

### Study design, patients, and treatment

This was a multicenter, randomized, open-label, phase 3 study, with a 12-week treatment period and a 8-week safety follow up. Eligible patients were aged ≥18, had a diagnosis of chronic plaque psoriasis, with a Psoriasis Area and Severity Index (PASI) score of 10 or higher (patients with a PASI score of 3 to less than 10 might also be enrolled if the investigator deemed them eligible for biologic therapy); had a static physician’s global assessment (sPGA) score of 3 or higher, and had at least 10% of body surface area (BSA) affected by psoriasis. Patients were excluded if they had pustular, erythrodermic, or guttate psoriasis, or drug-induced psoriasis, or other inflammatory diseases such as inflammatory bowel disease, uveitis, atopic dermatitis, etc., or active autoimmune diseases such as systemic sclerosis, systemic lupus erythematosus, etc., which might affect the efficacy or safety assessment. Patients were excluded if they had prior received biologics and biosimilars, including but not limited to: etanercept for less than 28 days; infliximab, adalimumab, or alefacept for less than 60 days; golimumab for less than 90 days; or any other biologics for less than five half-lives). Patients were excluded if they had received biologics targeting IL-17 or the IL-17 receptor, IL-12/IL-23, or IL-23 within 5 half-lives or 3 months prior to randomization (whichever was longer); however, those who had received IL-17 targeted therapy before this specified washout period were eligible for inclusion.

At baseline, patients were randomized 1:1 to receive subcutaneous injections of 80 mg of SSGJ-608 every 2 weeks (Q2W) after a starting dose of 160 mg at week 0 (608A group), or 160 mg of SSGJ-608 every 4 weeks (Q4W) (608B group). After the 12-week treatment period, patients underwent the safety follow-up at week 20 and completed the trial.

All patients provided written informed consent before study initiation. The study protocol adhered to the Declaration of Helsinki, International Conference for Harmonization guidelines, and other relevant regulations. The ethics committee at each site approved the protocol and all amendments.

### Assessments

Efficacy was assessed by the proportion of patients who achieved PASI75 at week 12 and the proportion who achieved sPGA 0 or 1 at week 12 as the co-primary endpoints. Secondary endpoints at week 12 included the proportion of patients who achieved PASI90, PASI100, or sPGA score of 0. Other efficacy assessments comprised: the proportion of patients who achieved a reduction of ≥4 points from baseline in the pruritus Numerical Rating Scale (NRS) score at week 12, and proportion of subjects achieving PASI 75, sPGA 0/1, PASI 90, PASI 100, and sPGA 0 at all assessment timepoints except week 12; changes of PASI score, BSA, the Dermatology Life Quality Index (DLQI), and pruritus NRS score were calculated from baseline to the end of the study; time to first achievement of PASI75, PASI90, and sPGA 0 or 1. Safety was evaluated by summarizing the occurrence of treatment-emergent adverse events (TEAEs) and serious adverse events (SAEs), which were categorized based on the Medical Dictionary for Regulatory Activities (MedDRA, version 27.1) and summarized by system organ class (SOC) and preferred terms (PT). Other safety outcomes included vital signs, physical examination, laboratory tests, electrocardiogram.

### Statistical analysis

Efficacy was assessed among all randomized patients, and safety was assessed among all randomized patients who received at least one dose of investigational drug. For the co-primary efficacy endpoints (PASI75 and sPGA 0/1 response rates at Week 12) and binary secondary endpoints (PASI90, PASI100, and sPGA 0 response rates at Week 12), the Clopper-Pearson exact method was used to calculate the 95% confidence intervals (CIs) within each treatment group. The primary statistical comparison between the two SSGJ-608 dose regimens was performed using the Cochran-Mantel-Haenszel (CMH) test, controlling for three pre-specified stratification factors: body weight (≥80 kg or <80 kg), baseline sPGA score (3 or 4), and prior exposure to IL-17/IL-17 receptor-targeted biologics (yes or no). Odds ratios (ORs) with 95% CIs were derived. Additionally, the Miettinen-Nurminen method was used to calculate the risk difference (RD) between groups and its 95% CI. Response rates over time at Weeks 4, 8, and 20 were summarized descriptively. For missing binary efficacy data, a pre-specified imputation rule was applied: if both the immediate preceding and following visits indicated a response, the missing value was imputed as ‘response’; otherwise, it was imputed as ‘No response’. For continuous endpoints (e.g., percent reduction in DLQI), descriptive statistics are presented. Time-to-event endpoints (e.g., time to first PASI75 response) were analyzed using the Kaplan-Meier method, with median times and 95% CIs reported. Adverse events will be coded using MedDRA (version 27.1), and summarized the number of cases and incidence rate by SOC, PT, and treatment group. Details of the duration, severity, causality assessment, actions taken, and outcomes of the adverse events were also presented. Laboratory test results and vital signs were summarized using statistical descriptions. Abnormal findings will be summarized using frequencies and percentages.

## Results

### Patients

**Figure 1:**
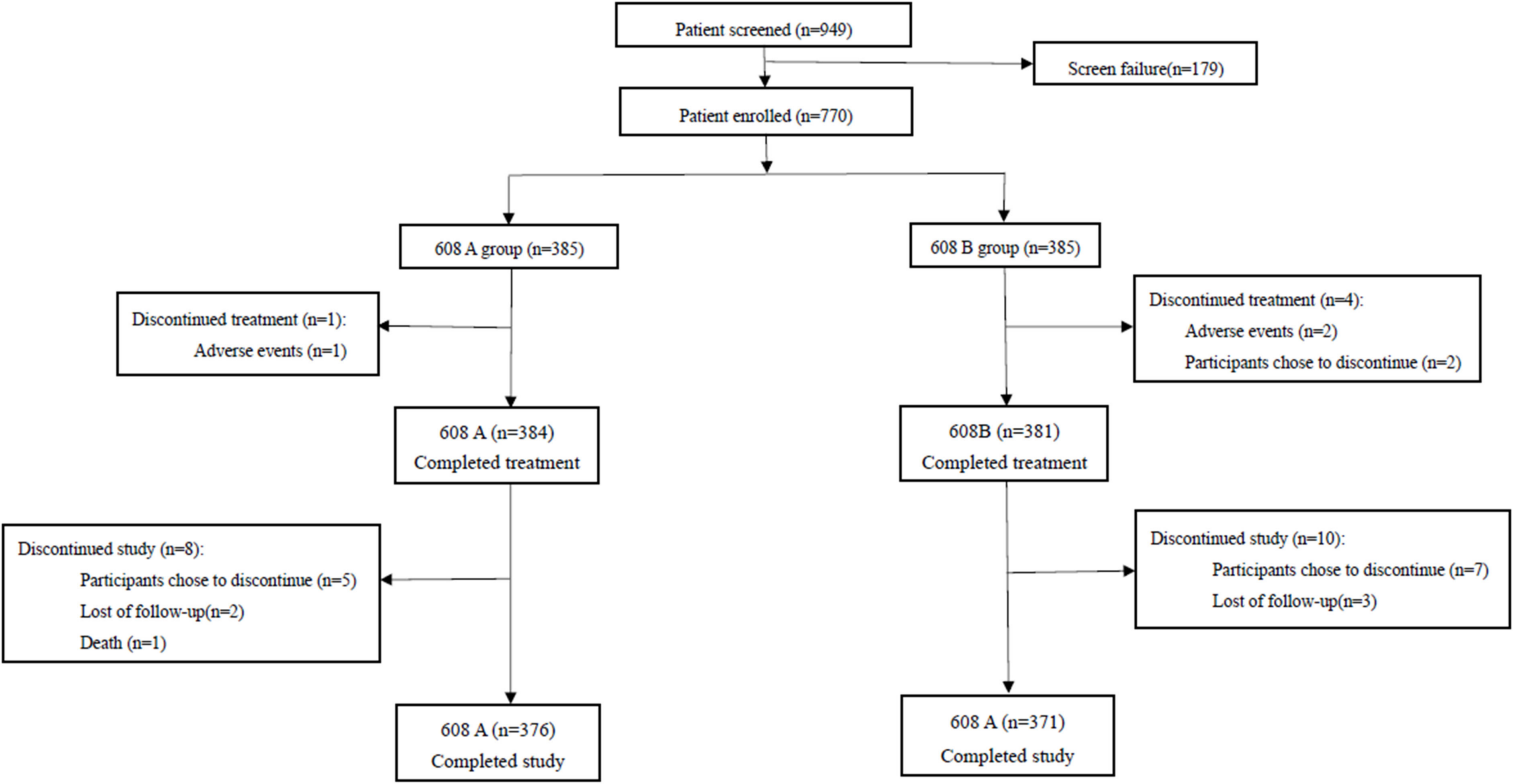
Patients disposition.

From April 2024 to January 2025, a total of 770 patients with moderate-to-severe plaque psoriasis were enrolled and randomized, of which 385 in the 608A group and 385 in the 608B group, respectively (**Figure 1**). One patient in the 608B group didn’t receive treatment after randomization. The baseline characteristics were consistent with the general population of patients with psoriasis. In total, 77.4% of the patients were male and the average age was 44.2 years. At baseline, the mean (Standard Deviation, SD) body surface area involvement was 23.88% (17.470); the mean PASI was 17.63 and the proportion of patients with sPGA of 3 or 4 were 65.7% and 34.3%, respectively. The mean baseline NRS score was 5.5 and the mean DLQI total score was 12.8 (**Table 1**).

**Table 1:** Demographics and baseline clinical characteristics.

Characteristics	608A (N = 385)	608B (N = 385)	Total (N = 770)
Age (years)	44.0 ± 13.77	44.4 ± 13.56	44.2 ± 13.66
Range	18-76	18-74	18-76
Sex, n (%)			
Male	306 (79.5)	290 (75.3)	596 (77.4)
Female	79 (20.5)	95 (24.7)	174 (22.6)
Weight(kg)	74.32 ± 14.853	73.59 ± 15.044	73.96 ± 14.944
Range	41.5-130.0	43.1-125.9	41.5-130.0
Duration of Psoriasis (years)	10.616 ± 10.7002	11.848 ± 10.6288	11.231 ± 10.6754
PASI	17.50 ± 9.374	17.76 ± 9.912	17.63 ± 9.641
sPGA, n (%)			
3	253 (65.7)	253(65.7)	506 (65.7)
4	132(34.3)	132(34.3)	264 (34.3)
BSA (%)	23.89 ± 17.247	23.87 ± 17.713	23.88 ± 17.470
Pruritus NRS	5.4 ± 2.56	5.5 ± 2.51	5.5 ± 2.53
DLQI	13.0 ± 7.17	12.7 ± 7.23	12.8 ± 7.20

Data are n (%) or mean ± SD.

PASI, Psoriasis Area and Severity Index; sPGA, static Physician’s Global Assessment; BSA, body surface area; NRS, Numerical Rating Scale; DLQI, Dermatology Life Quality Index.

### Efficacy

**Figure 2:**
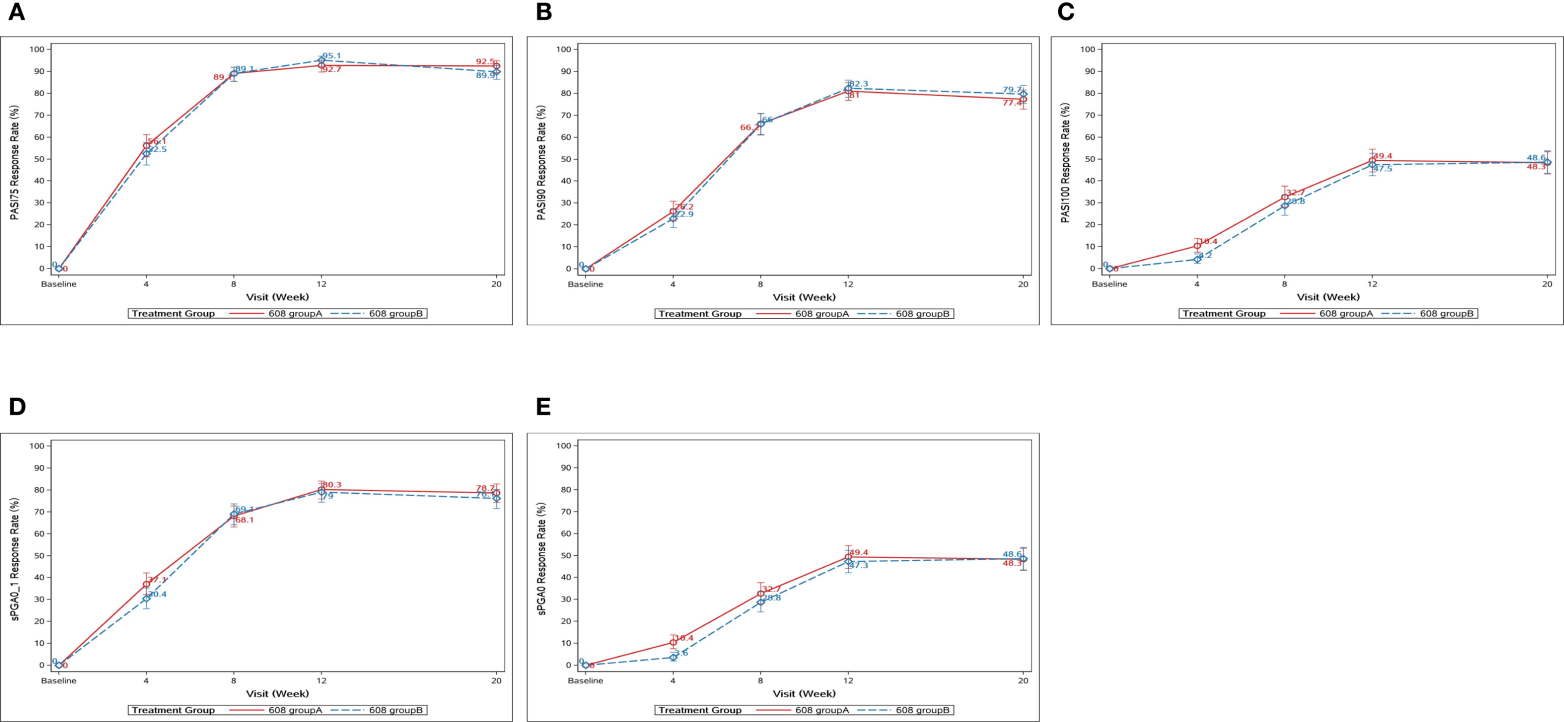
Clinical response over time in patients with moderate to severe plaque psoriasis. Proportion of patients achieving **(A)** PASI75 **(B)** PASI90 **(C)** PASI100 **(D)** sPGA 0or1 **(E)** sPGA 0. PASI, Psoriasis Area and Severity Index; sPGA, static Physician’s Global Assessment.

Following 12 weeks of SSGJ-608 treatment, the proportion of patients achieving PASI75 was 92.7% (357/385) in the 608A group and 95.1% (366/385) in the 608B group, respectively; the proportion of patients achieving sPGA 0 or 1 in each group was 80.3% (309/385) and 79.0% (304/385), respectively. At week 12, 81.0% (312/385) of patients in the 608A group and 82.3% (317/385) in the 608B group achieved PASI90, respectively; 49.4% (190/385) and 47.5% (183/385) in each group achieved PASI100, respectively; sPGA score of 0 was achieved by 49.4% (190/385) of patients and 47.3% (182/385) in each group, respectively (**Table 2**; **Figure 2**).

**Table 2:** Primary and secondary endpoints.

Efficacy endpoints	608A (N = 385)	608B (N = 385)
Primary endpoints		
PASI 75 at week 12	357 (92.7)	366 (95.1)
Risk difference (95%CI)	-2.8(-6.59, 1.00)	
Odds ratio (95%CI)	0.66(0.36, 1.21)	
sPGA 0 or 1 at week 12	309 (80.3)	304 (79.0)
Risk difference (95%CI)	1.4 (-4.15, 6.92)	
Odds ratio (95%CI)	1.08 (0.76, 1.54)	
Secondary endpoints		
PASI 90 at week 12	312 (81.0)	317 (82.3)
Risk difference (95%CI)	-0.6 (-5.99, 4.89)	
Odds ratio (95%CI)	0.91 (0.63, 1.32)	
PASI100 at week 12	190 (49.4)	183 (47.5)
Difference (95%CI)	1.6 (-5.22, 8.50)	
Odds ratio (95%CI)	1.07 (0.81, 1.42)	
sPGA 0 at week 12	190 (49.4)	182 (47.3)
Risk difference (95%CI)	1.9 (-4.97, 8.75)	
Odds ratio (95%CI)	1.08 (0.82, 1.44)	

PASI, Psoriasis Area and Severity Index; sPGA, static Physician’s Global Assessment. CI, confidence interval.

PASI 75 response rate was 56.1% (216/385) in the 608A group and 52.5% (202/385) in the 608B group at week 4; this rate increased to 89.1% (343/385) and 89.1% (343/385) at week 8, and further improved to 92.5% (356/385) and 89.9% (346/385) by week 20, respectively. sPGA 0 or 1 was achieved by 37.1% (143/385) in the 608A group and 30.4% (117/385) in the 608B group at week 4; this rate increased to 68.1% (262/385) and 69.1% (266/385) at week 8, and further improved to 78.7% (303/385) and 76.1% (293/385) by week 20, respectively. PASI 90 response rate was 26.2% (101/385) in the 608A group and 22.9% (88/385) in the 608B group at week 4; this rate increased to 66.2% (255/385) and 66.0% (254/385) at week 8, and further improved to 77.4% (298/385) and 79.7% (307/385) by week 20, respectively. PASI100 response rate was 10.4% (40/385) in the 608A group and 4.2% (16/385) in the 608B group at week 4; this rate increased to 32.7% (126/385) and 28.8% (111/385) at week 8, and further improved to 48.3% (186/385) and 48.6% (187/385) by week 20, respectively. sPGA score of 0 was 10.4% (40/385) in the 608A group and 3.6% (14/385) in the 608B group at week 4; this rate increased to 32.7% (126/385) and 28.8% (111/385) at week 8, and further improved to 48.3% (186/385) and 48.6% (187/385) by week 20, respectively (**Supplementary Table 1**; **Figure 2**).

Besides, of the patients who had a pruritus NRS score ≥4 at baseline, 82.3% (242/294) of patients in the 608A group and 78.9% (235/298) in the 608B group achieved a reduction in pruritus NRS score of ≥4 at week 12 (**Supplementary Table 2**). Regarding quality of life, the mean DLQI score reduction of 79.63% in the 608A group and 75.47% in the 608B group at week 12 compared to baseline, respectively; and the reduction was 83.55% in the 608A group and 76.47% in the 608B group at week 20 compared to baseline, respectively.

At week 12, in the subgroup of patients who had received IL-17 targeted therapy before, 77.8% (42/54) of patients in the 608A group and 74.0% (37/50) in the 608B group achieved PASI90, respectively; 55.6% (30/54) and 44.0% (22/50) in each group achieved PASI100, respectively; sPGA 0 was achieved by 55.6% (30/54) and 44.0% (22/54) in each group, respectively (**Supplementary Table 3**).

### Safety

A total of 769 patients were enrolled in the safety population, and one patient in the 608B group didn’t receive treatment after randomization was excluded from this population. Overall, 64.2% (247/385) of patients in the 608A group and 62.8% (241/384) in the 608B group experienced TEAEs. SAEs were reported in 9(2.3%) patients in the 608A group and 5(1.3%) patients in the 608B group, and all these SAEs were assessed as definitely unrelated or possibly unrelated to the investigational drug. Discontinuations due to TEAEs were low, occurring in only 1 (0.3%) patient in the 608A group and 2(0.5%) patients in the 608B group, with one case of generalized rash and one case of eczema assessed as related to the investigational drug (**Table 3**).

**Table 3:** Overview of adverse events.

Type of Events	608A (N = 385)	608B (N = 384)	Total (N = 769)
Any treatment-emergent adverse event	247 (64.2)	241 (62.8)	488 (63.5)
Serious adverse event	9 (2.3)	5 (1.3)	14 (1.8)
TEAE leading to discontinuation	1 (0.3)	2 (0.5)	3 (0.4)
TEAE leading to death	1 (0.3)	0	1 (0.1)
Common adverse event			
Hypertriglyceridemia	18 (4.7)	22 (5.7)	40 (5.2)
Upper respiratory tract infection	21 (5.5)	17 (4.4)	38 (4.9)
Hyperuricemia	20 (5.2)	14 (3.6)	34 (4.4)
Increased alanine aminotransferase	21 (5.5)	12 (3.1)	33 (4.3)
Hypercholesterolemia	15 (3.9)	13 (3.4)	28 (3.6)
Hyperlipidemia	13 (3.4)	14 (3.6)	27 (3.5)
Blood uric acid increased	15 (3.9)	11 (2.9)	26 (3.4)
Eczema	14 (3.6)	12 (3.1)	26 (3.4)
Sinus bradycardia	12 (3.1)	11 (2.9)	23 (3.0)
Blood creatine phosphokinase increased	16 (4.2)	7 (1.8)	23 (3.0)
Blood glucose increased	12 (3.1)	11 (2.9)	23 (3.0)

Data are n (%).

TEAE, treatment-emergent adverse event.

The most common TEAEs were hypertriglyceridemia (5.2%), upper respiratory tract infection (4.9%), hyperuricemia (4.4%), increased alanine aminotransferase (4.3%) and hypercholesterolemia (3.6%). The incidence of drug-related infections and infestations was 6.0% in the 608A group and 7.6% in the 608B group, with the upper respiratory tract infection being most common (2.6% in the 608A group and 3.1% in the 608B group, respectively). The incidence of injection site reaction (ISR) was 2.9%), and no cases of either inflammatory bowel disease (IBD) or candidal infections were reported with SSGJ-608 in this study.

## Discussion

In this study, SSGJ-608 was highly effective for treating patients with moderate-to-severe plaque psoriasis, as shown by the achievement of coprimary endpoints and secondary endpoints, at 80mg Q2W (with a 160mg starting dose at Week 0) and 160mg Q4W in a larger population. The clinical response to SSGJ-608 was rapid, with approximately 56.1% of patients in the 608A group and 52.5% in the 608B group achieving PASI75 at week 4, after one or two doses of SSGJ-608. The clinical outcomes demonstrated substantial improvement, with 92.7% and 95.1% of patients in each group achieving PASI75, and 80.3% and 79.0% in each group achieving sPGA 0 or 1 at week 12, respectively. Moreover, SSGJ-608 also achieved high rates of PASI90 (77.8% vs.74.0%) and PASI100 (55.6% vs. 44.0%) in the subgroup of patients previously treated with anti-IL-17 therapy in both treatment groups at week 12. The clinical responses of SSGJ-608 were comparable in two dosing schedules, and the key efficacy data aligned with the results from the pivotal phase 3 trial, further reinforcing the robustness of SSGJ-608.

SSGJ-608 was associated with superior efficacy in achieving high-level clearance, as measured by PASI90 and PASI100 responses. At week 12, PASI90 was achieved by 80.1% and 82.3% in the two groups, and PASI100 response rate was 49.4% and 47.5%, respectively. Although cross-trial comparisons require caution,reported week 12 PASI90 rates with secukinumab is 39.1% to 59.2%; PASI100 rates at week12 with secukinumab, ixekizumab and vunakizumab are 12.8% to 28.6%, 33.6 to 35.3%, and 36.6%, respectively (16, 18, 19). High levels of PASI100 responses with SSGJ-608 may raise treatment goals for patients, as complete skin clearance translated into the greatest benefits in health-related quality of life and patient-perceived symptoms (20). Furthermore, the proportions of patients achieving sPGA 0 in the two groups (49.4% and 47.3%) were comparable to the PASI100 response rates, with both endpoints signifying complete skin clearance. The clinical responses of SSGJ-608 at 80mg Q2W and 160mg Q4W in a larger population showed potential superior efficacy in achieving high-level clearance. The ability of SSGJ-608 to sustain high levels of complete or near-complete skin clearance is critical for ensuring long-term treatment success and improving patients’ quality of life.

At week 20 (10 to 12 weeks after last dose), PASI90 response rates were 77.4% and 79.7% in the two groups, and PASI100 response rates were 48.3% and 48.6%, respectively. Comparatively, reported week 60 (12 weeks after last dose) PASI90 and PASI100 rates with xeligekimab (75.5% and 50.6%) were similar with SSGJ-608 (21); PASI100 rate at week 20 (8 weeks after last dose) with HB0017 (150mg, Q4W) was 37%, which was lower than SSGJ-608 (22). The sustained clinical response of SSGJ-608 after treatment withdrawal represents a key advantage for patients who may require a therapy pause. Historically, maintaining a good response with less regular dosing schedules might have been challenging with some treatments, and many patients with psoriasis who take biologics lose response over time or frequently switch treatments to maintain clinical responses (11, 23). This underscores the value of SSGJ-608’s durable post-withdrawal effect. Notably, in the subgroup of patients previously treated with anti-IL-17 therapy, SSGJ-608 also achieved high rates of PASI90 (77.8% vs.74.0%) and PASI100 (55.6% vs. 44.0%) in both treatment groups at week 12. These findings suggest a potential clinical benefit of SSGJ-608 in managing psoriasis patients who have lost response to prior biologics or who need frequent regimen changes to maintain efficacy.

Overall, SSGJ-608 was well tolerated in the long term, with a low incidence of TEAEs leading to discontinuation and no increased risk of TEAEs with increased duration of exposure. The safety profile of SSGJ-608 was generally consistent with that of other available IL-17A inhibitors, and no new safety signals were identified (16, 18, 24). The incidence of TEAEs was similar between the two recommended doses of SSGJ-608, indicating that a more frequent dosing regimen did not increase the risk of adverse events.

The most frequently reported adverse events in this study were hypertriglyceridemia, upper respiratory tract infection, hyperuricemia, increased alanine aminotransferase and hypercholesterolemia, most of which were metabolism-related and infectious events. The upper respiratory tract infections and hyperuricemia observed with SSGJ-608 are also common in Chinese patients with psoriasis who received secukinumab or ixekizumab (18, 25, 26). The overall incidence of infections and infestations was 14.4% and 6.8% was drug-related, which is much lower than in ixekizumab (55.2%) (16). the incidence of injection site reaction (ISR) was extremely low (2.9%), compared to ixekizumab (10.4%) (16), vunakizumab (10.9%) (19) and xeligekimab (9.6%) (21). Importantly, no cases of either inflammatory bowel disease (IBD) or candidal infections were reported with SSGJ-608 in this study. Both events are recognized risks of IL-17 inhibition, a pathway crucial for mucosal homeostasis and antifungal defense (16, 18, 24, 27). These data indicated that SSGJ-608 had a potential clinical advantage in minimizing injection-related discomfort, which may contribute to better patient adherence. Moreover, by lowering the risk of significant complications such as cardiovascular and inflammatory diseases, SSGJ-608 may contribute to extended patient survival and enhanced quality of life.

The study has several limitations. First, as only Chinese patients were enrolled, the generalizability of the treatment outcomes to other ethnic groups remains to be established. Second, the absence of a control group, such as active comparator, limits the conclusive assessment of the therapeutic benefits of SSGJ-608 in plaque psoriasis patients, further investigation is warranted to evaluate the comparative efficacy and safety of SSGJ-608 relative to other approved biologics for psoriasis.

In summary, this study showed rapid, substantial, and durable levels of skin clearance with SSGJ-608 in patients with moderate to severe psoriasis in both 80mg Q2W (with a 160mg starting dose at Week 0) and 160mg Q4W in a larger population, which was reflected in high proportions of complete skin clearance. SSGJ-608 also achieved high clinical response rates in patients previously treated with anti-IL-17 therapy in both dosing schedules. Safety findings in this patient population were consistent with other same-target drugs, and no new risk signals were found for SSGJ-608. This study featured a large sample size and sufficient exposure, further validating both the efficacy and safety profile of SSGJ-608. These findings suggest that SSGJ-608 has the potential to ensure long-term therapeutic success and improve patient quality of life, while also presenting a lower risk of certain significant complications.

## Data Availability

The raw data supporting the conclusions of this article will be made available by the authors, without undue reservation.

## Supplementary material

Supplementary Table 1 Clinical response over time except week 12.

Supplementary Table 2 Patients who had a pruritus NRS score ≥4 at baseline who achieved.

Supplementary Table 3 Clinical response at week 12 in patients who had received IL-17 targeted therapy before.
