# Supplementary material for: SSGJ-608 in moderate-to-severe plaque psoriasis: a multicenter, randomized, open-label, phase 3 study
Source: Front Immunol. 2026 Jun 9;17:1810418. doi: 10.3389/fimmu.2026.1810418 (PMC13286926; doi:10.3389/fimmu.2026.1810418)
Supplement: Supplementary Table 1 — Clinical response over time except week 12. [file Table1.docx]

Supplement materials

Table S1 Clinical response over time except week 12.

|  | 608A  (N=385) | 608B  (N=385) |
| --- | --- | --- |
| PASI75 response  Response at week 4  95%CI | 216(56.1)  50.99, 61.13 | 202(52.5)  47.35, 57.55 |
| Response at week 8  95%CI  Response at week 20  95%CI | 343(89.1)  85.54, 92.02  356(92.5)  89.36, 94.90 | 343(89.1)  85.54, 92.02  346(89.9)  86.41, 92.70 |
| sPGA0/1 response  Response at week4  95%CI  Response at week 8  95%CI  Response at week 20  95%CI | 143(37.1)  32.30, 42.18  262(68.1)  63.14, 72.68  303(78.7)  74.27, 82.69 | 117(30.4)  25.83, 35.25  266(69.1)  64.21, 73.67  293(76.1)  71.52, 80.28 |
| PASI90 response  Response at week 4  95%CI  Response at week 8  95%CI  Response at week 20  95%CI  PASI100 response  Response at week 4  95%CI  Response at week 8  95%CI  Response at week 20  95%CI  sPGA0 response  Response at week 4  95%CI  Response at week 8  95%CI  Response at week 20  95%CI | 101(26.2)  21.91, 30.93  255 (66.2)  61.27, 70.95  298(77.4)  72.89, 81.49  40(10.4)  7.53, 13.88  126(32.7)  28.06, 37.66  186(48.3)  43.22, 53.43  40(10.4)  7.53, 13.88  126(32.7)  28.06, 37.66  186(48.3)  43.22, 53.43 | 88(22.9)  18.76, 27.38  254(66.0)  61.00, 70.70  307(79.7)  75.37, 83.64  16(4.2)  2.39, 6.66  111(28.8)  24.35, 33.64  187(48.6)  43.48, 53.69  14(3.6)  2.00, 6.03  111(28.8)  24.35, 33.64  187(48.6)  43.48, 53.69 |

*PASI, Psoriasis Area and Severity Index; sPGA, static Physician’s Global Assessment.*

*CI:* *confidence interval*
