# Supplementary material for: SSGJ-608 in moderate-to-severe plaque psoriasis: a multicenter, randomized, open-label, phase 3 study
Source: Front Immunol. 2026 Jun 9;17:1810418. doi: 10.3389/fimmu.2026.1810418 (PMC13286926; doi:10.3389/fimmu.2026.1810418)
Supplement: Supplementary Table 2 — Patients who had a pruritus NRS score ≥4 at baseline who achieved. [file Table2.docx]

Supplement materials

Table S2 Time to first response of PASI75, PASI90 and sPGA 0/1

during the double-blind induction treatment period

|  | 608A  (N=184) | 608B  (N=183) |
| --- | --- | --- |
| Time to first response of PASI75  Median  95%CI  Time to first response of PASI90  Median  95%CI  Time to first response of sPGA0/1  Median  95%CI | 31.0  30.00,32.00  57.0  57.00, 58.00  57.0  56.00, 57.00 | 32.0  30.00,54.00  57.0  57.00, 58.00  57.0  57.00, 58.00 |

*PASI, Psoriasis Area and Severity Index; sPGA, static Physician’s Global Assessment.*

*CI:* *confidence interval*
