# Supplementary material for: SSGJ-608 in moderate-to-severe plaque psoriasis: a multicenter, randomized, open-label, phase 3 study
Source: Front Immunol. 2026 Jun 9;17:1810418. doi: 10.3389/fimmu.2026.1810418 (PMC13286926; doi:10.3389/fimmu.2026.1810418)
Supplement: Supplementary Table 3 — Clinical response at week 12 in patients who had received IL-17 targeted therapy before. [file Table3.docx]

Supplement materials

Table S3 Patients who had a NRS score ≥4 at baseline who achieved

a reduction in NRS score of ≥4 at week 12

|  | 608A  (N=294) | 608B  (N=298) |
| --- | --- | --- |
| Patients who had a NRS score ≥4 at baseline  Reduction in NRS score at week12  Response, n (%)  95%CI  Risk difference vs. placebo(95%CI)  Odds ratio vs. placebo(95%CI) | 242(82.3)  77.46, 86.50  3.3(-3.03, 9.59)  1.27(0.84) | 235(78.9)  73.78, 83.76 |

*NRS: Numerical Rating Scale*
